# PfCDPK1 mediated signaling in erythrocytic stages of *Plasmodium falciparum*

**DOI:** 10.1038/s41467-017-00053-1

**Published:** 2017-07-05

**Authors:** Sudhir Kumar, Manish Kumar, Roseleen Ekka, Jeffrey D. Dvorin, Aditya S. Paul, Anil K. Madugundu, Tim Gilberger, Harsha Gowda, Manoj T. Duraisingh, T. S. Keshava Prasad, Pushkar Sharma

**Affiliations:** 1 Eukaryotic Gene Expression laboratory, National Institute of Immunology, New Delhi, 110067 India; 2 Institute of Bioinformatics, Bangalore, Karnataka 560066 India; 3 Manipal University, Madhav Nagar, Manipal, Karnataka 576104 India; 4 Harvard TH Chan School of Public Health, Harvard University, Boston, Massachusetts 02115 USA; 5 Boston Children’s Hospital and Harvard Medical School, Boston, Massachusetts 02115 USA; 6 Centre for Bioinformatics, Pondicherry University, Puducherry, 605014 India; 7 Bernhard Nocht Institute for Tropical Medicine and Centre for Structural Systems Biology, Hamburg, 20359 Germany; 8 YU-IOB Center for Systems Biology and Molecular Medicine, Yenepoya University, Mangalore, 575018 India; 9 Proteomics and Bioinformatics Laboratory, Neurobiology Research Centre, National Institute of Mental Health and Neuro Sciences, Bangalore, Karnataka 560029 India

## Abstract

Calcium Dependent Protein Kinases are key effectors of calcium signaling in malaria parasite. PfCDPK1 is critical for asexual development of *Plasmodium falciparum*, but its precise function and substrates remain largely unknown. Using a conditional knockdown strategy, we here establish that this kinase is critical for the invasion of host erythrocytes. Furthermore, using a multidisciplinary approach involving comparative phosphoproteomics we gain insights into the underlying molecular mechanisms. We identify substrates of PfCDPK1, which includes proteins of Inner Membrane Complex and glideosome-actomyosin motor assembly. Interestingly, PfCDPK1 phosphorylates PfPKA regulatory subunit (PfPKA-R) and regulates PfPKA activity in the parasite, which may be relevant for the process of invasion. This study delineates the signaling network of PfCDPK1 and sheds light on mechanisms via which it regulates invasion.

## Abstract (web summary)

Calcium dependent protein kinase 1 (CDPK1) plays an important role in asexual development of *Plasmodium falciparum*. Using phosphoproteomics and conditional knockdown of CDPK1, the authors here identify CDPK1 substrates and a cross-talk between CDPK1 and PKA, and show the role of CDPK1 in parasite invasion.

## Introduction

Protozoan parasite *Plasmodium falciparum* is a major causative agent of pernicious human malaria, which infects the mosquito and the human host. *Plasmodium* infects two different cell types in the human host; hepatocytes and the erythrocytes and encounters diverse host environments, which it successfully survives to propagate. The asexual development of the parasite involves invasion of host RBCs by the parasite followed by its intraerythrocytic development, multiplication and egress to invade fresh RBCs, which results in clinical manifestations and pathogenesis of disease. While it has become increasingly clear that the subtle regulation of parasite developmental processes occurs via signaling events, the underlying pathways are poorly understood at the molecular level. Calcium signaling is a central player that regulates almost all developmental stages of the parasite^1, 2^. The parasite entraps calcium, which it possibly acquires during invasion^3^, in intracellular stores and uses it judiciously during the course of its development. Phospholipase C^4^, as well as cADP-ribose pathways^5^ have been shown to exist in the parasite and are involved in host cell invasion and sexual differentiation^4, 6^.

The malarial parasite possesses unique sets of calcium effectors Calcium Dependent Protein Kinases (CDPKs), which are absent from the host but regulate processes in *Plasmodium* and related apicomplexan *Toxoplasma gondii* ^1, 2^. While the parasite seems to lack the classical Calmodulin dependent protein kinases (CamKs), CDPKs possess a Calmodulin Like Domain at their C-terminus via which calcium dependent activation of the enzyme occurs^7^. PbCDPK4 regulates sexual differentiation of the rodent parasite *P. berghei* and PfCDPK5 guides the egress of merozoites from the host erythrocyte^8^. CDPKs regulate important processes in *T. gondii* as well. For instance, TgCDPK1 is involved in microneme secretion triggered by calcium^9^. While PbCDPK1 is not essential for *P. berghei* asexual development^10^, independent reports have suggested that PfCDPK1 is refractory to gene disruption^11, 12^, indicating that it may be indispensable for asexual blood stage development of *P. falciparum*. Insights into the putative role of PfCDPK1 in *P. falciparum* have come from studies using a pharmacological inhibitor purfalcamine^11^, and peptide inhibitors^13^ which have suggested that PfCDPK1 may be involved in egress from the RBC and/or invasion. Since these inhibitors and other tools are likely to “hit” other targets, the specific function of CDPK1 in *P. falciparum* life cycle remains largely unknown and a genetic approach was needed to specifically determine its role in parasite biology.

We have used a conditional “knockdown” approach to dissect the role of PfCDPK1 in the process of host RBC invasion. Quantitative phosphoproteomics was employed for the identification of potential PfCDPK1 targets in the parasite to gain insights into its putative role in this process. PfCDPK1 may contribute to the process of invasion by regulating key parasite proteins, which include proteins of the inner membrane complex (IMC) and cAMP signaling module. Present studies also identify molecular interactions via which calcium and cAMP pathways may cross-talk in the parasite.

## Results

### Conditional knockdown of PfCDPK1 in *P. falciparum*

**Fig. 1:**
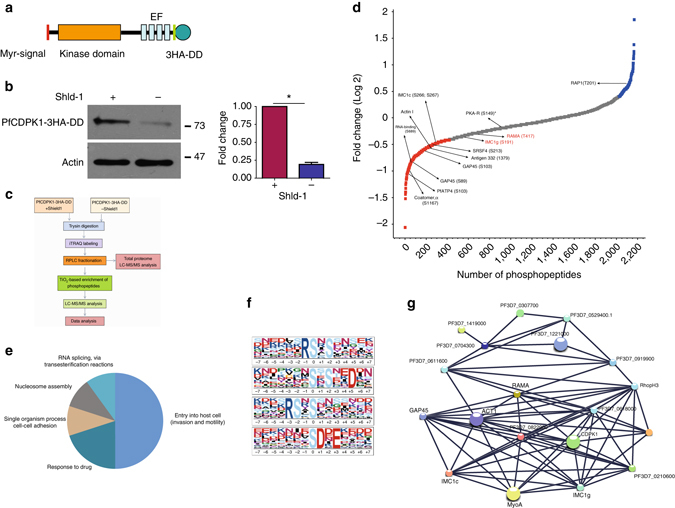
Identification of putative PfCDPK1 substrates and interacting proteins. **a** PfCDPK1-3HA-DD parasites were generated by fusing the C-terminus of PfCDPK1, with an FKBP Death Domain (DD) and HA tag (Supplementary Fig. 1). PfCDPK1 possesses a N-terminal myristoylation and palmitoylation motif (red) and a C-terminal calmodulin like domain. The DD domain targets PfCDPK1 to proteasomic degradation in the absence of its ligand Shld-1. **b** PfCDPK1-3HA-DD parasites were cultured in the presence (+) of Shld-1. At the ring stage, parasites were split and Shld-1 was removed (−) from one half of the cultures. The cultures were harvested at the schizont stage and western blotting was performed using anti-HA antibody to detect the expression of PfCDPK1-3HA-DD. Actin was used as the loading control. *Right Panel*, Densitometry was performed for PfCDPK1 bands and was normalized with respect to the loading control (Mean±s.e.m., *n=3, *p*<0.0001, *t*-test). **c** Schematic illustrating the work flow followed for quantitative proteomics and phosphoproteomics studies from Shld-1 treated (+) or deprived parasites (−) (for details see Methods). **d** The phosphorylation ratios of phosphopeptides identified from PfCDPK1-3HA-DD schizonts cultured in the presence or absence of Shld-1 was normalized with respect to total protein. The ratios for all phosphopeptides from various replicates are provided in Supplementary Data 1. The S-curve for one of the replicates is provided and some of the significantly altered phosphorylation sites belonging to key proteins (Supplementary Data 2) are indicated. Two key proteins that were marginally higher than the cut-off in the replicate represented here but were significantly altered in two other replicates (Supplementary Data 2) are lettered in red. PfPKA-R-S149, which was altered in only one replicate (Supplementary Data 1), is indicated by *. **e** Proteins that exhibited reduced phosphorylation upon PfCDPK1 depletion were subjected to Gene Ontology enrichment analysis. Biological process of “entry into host cell” that consists of proteins involved in invasion and motility was found to be significantly enriched (Supplementary Data 2). **f** Distribution of amino acids surrounding the S/T in proteins that exhibited significant reduction in phosphorylation (Supplementary Data 2). The four major motifs that emerged from Motif-X analysis are indicated. **g** Protein–protein interaction was predicted between differentially phosphorylated proteins using STRING resource. The analysis exhibited high confidence protein–protein interactions between the candidate genes (**d**, Supplementary Data 3)

To elucidate the function of PfCDPK1 and identify its putative substrates, we generated parasites in which PfCDPK1 expression was down regulated. Since PfCDPK1 is refractory to gene disruption^11, 12^, a FKBP destabilization domain (DD) strategy was used to conditionally regulate its expression in *P. falciparum* ^14^. The DD-fusion proteins are unstable in the absence of the DD ligand Shield-1 (Shld-1), which allows conditional knockdown of fused proteins. Using a single crossover strategy, PfCDPK1 was fused to the triple HA- tag followed by a DD domain at its C-terminus (Fig. 1a, Supplementary Fig. 1) to obtain PfCDPK1-3HA-DD parasites, which were cultured in the presence of Shld-1. The removal of Shld-1 resulted in ~70% reduction of PfCDPK1 expression as quantified by western blotting (Fig. 1b).

### Identification of potential PfCDPK1 substrates

In order to elucidate the mechanism via which PfCDPK1 interplays with cellular processes of the parasite, it was important to globally identify proteins that undergo PfCDPK1-dependent phosphorylation in the parasite to unravel CDPK1 signaling pathways. The phosphoproteome of PfCDPK1-3HA-DD parasites was compared in the presence or absence of Shld-1. During asexual blood stage reproduction, PfCDPK1 exhibits maximum expression in late schizont stages and merozoites^15^. Therefore, it was pertinent to use these stages for phosphoproteomics. Shld-1 was removed at the ring stage and late schizont lysates were prepared for phosphoproteomic analysis. A mass spectrometry-based quantitative phosphoproteomic and proteomic approach involving iTRAQ labeling to measure differences in the phosphorylation state of proteins upon PfCDPK1 knockdown was employed (Fig. 1c). These studies resulted in identification of 2057 unique phosphosites from 1233 *P. falciparum* proteins (Fig. 1d, Supplementary Data 1). Several of these peptides exhibited differential phosphorylation upon PfCDPK1 knockdown achieved by Shld-1 removal (Fig. 1d, Supplementary Data 1).

**Fig. 2:**
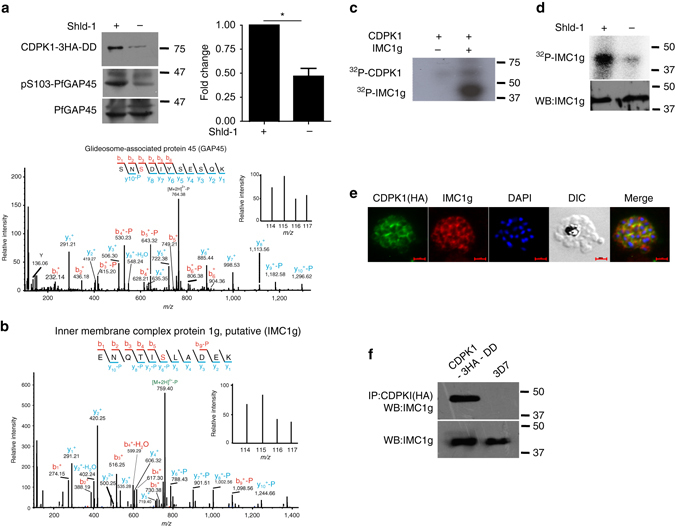
PfCDPK1 interacts and phosphorylates proteins of IMC and glideosome complex. **a** The phosphorylation status of PfGAP45 at S103 was assessed after Shld-1 withdrawal from PfCDPK1-3HA-DD parasites. Parasite lysates were prepared from late schizont/segmenter and Western blotting was performed using antibody against PfGAP45 or its form which is phosphorylated at S103^15^. *Right Panel*, Densitometry was performed for pS103-PfGAP45 bands and was normalized with respect to the WT PfGAP45 (Mean±s.e.m., *n=3, *p*<0.0001, *t*-test). *Lower Panel*, MS/MS spectra for S103 containing phosphopeptide is provided, which indicates differential phosphorylation at this site. **b** MS/MS spectra of phosphopeptides from IMC1g protein, which was differentially phosphorylated upon PfCDPK1 depletion. **c** Recombinant 6xHis-PfCDPK1 was used to phosphorylate recombinant 6xHis-IMC1g in a kinase assay using γ^32-^P[ATP]. The assay mix was electrophoresed on a SDS-PAGE gel and phosphate incorporation was observed by autoradiography. **d** PfCDPK1-3HA-DD parasites were synchronized and cultured in the presence or absence of Shld-1. Parasites were labeled with ^32^P-orthophosphate and IMC1g was immunoprecipitated. IMC1g-IP was subjected to phosphorimaging and parasite lysate was used for Western blotting with anti-IMC1g. **e** IFA was performed on PfCDPK1-3HA-DD parasites using anti-HA antibody to detect CDPK1 and anti-IMC1g antisera. Scale bar is 2 µm. **f** PfCDPK1 was immunoprecipitated from PfCDPK1-3HA-DD or 3D7 (control) parasites using anti-HA antibody. Western blotting was performed on both PfCDPK1-IP and total lysate using anti-IMC1g antisera

Proteins that exhibited >0.75 fold change in phosphorylation in at least two replicates at specific residues upon PfCDPK1 ablation were considered as significant (Supplementary Data 1). This cutoff range was defined by using the PfCDPK1-dependent phosphorylation of S103 in PfGAP45 as an internal standard, as this protein was previously demonstrated to be phosphorylated by PfCDPK1 in vitro^15^. This glideosome-associated protein is expressed in late stage parasites and showed a ~0.75 fold reduction in phosphorylation at S103 upon PfCDPK1 knockdown, which could be detected using a S103 specific phospho-antibody (Fig. 2a, see below).

Since PfCDPK1 is a kinase, the proteins that exhibited significant reduced phosphorylation upon its depletion were of specific interest. In all, 79 sites from 64 proteins exhibited significant decrease in phosphorylation in at least two replicates (Fig. 1d, Supplementary Data 2). Motif analysis of hypophosphorylated peptides showed overrepresentation of certain conserved amino acid sequences spanning the altered phosphorylation sites. Interestingly, most represented motifs comprised of basic amino acids with a preference for an R (or K) preceding the target S/T (Fig. 1f, Supplementary Data 2). These data agree well with previously identified preferred motifs for CDPK phosphorylation^16^. In addition, motifs in which target S/T were followed by acidic D/E residues were also found to be modulated (Fig. 1f, Supplementary Data 2).

Gene Ontology (GO) analysis suggested that a number of differentially phosphorylated proteins may be involved in invasion and/or motility (Fig. 1e, Supplementary Data 2). Several ribosomal proteins, proteins involved in RNA metabolism and processing, were also part of this group, but they are unlikely to be a direct PfCDPK1 targets. Surprisingly, the low molecular weight rhoptry complex containing RAMA and RAP1 also appeared to be modulated by PfCDPK1.

The regulatory subunit of cAMP dependent Protein Kinase (PfPKA-R) exhibited a change in its phosphorylation status at S149, which was just above the indicated threshold in phosphoproteomic analysis (Fig. 1d, Supplementary Data 1). Importantly, this phosphorylation site was part of a motif closely related to PfCDPK1 target motifs mentioned above. Since PfPKA-R is a key signaling molecule of the cAMP pathway, which our preliminary studies had implicated in PfCDPK1 function, it was also validated and pursued for further studies (described below).

Interestingly, a large number of putative target proteins were predicted or known to be associated with the IMC and the parasite pellicle (Fig. 1e, Supplementary Fig. 2, Supplementary Data 2), a membranous structure that underlies the plasma membrane. Given the membrane localization of PfCDPK1, which is facilitated by its N-terminus lipid modification and its previously reported interaction with IMC proteins in the membrane^17, 18^, these findings corroborated well and raised the possibility of these targets in close proximity of PfCDPK1.

We predicted the likelihood of protein–protein interactions (PPI) among the differentially phosphorylated proteins identified in our study from STRING database (Fig. 1g, Supplementary Data 3), which is a resource of PPI that have emerged either from direct experiments reported in the literature and predicted on the basis of co-expression and gene arrangement in the genome^19^. STRING has been used successfully to predict PPI in *Plasmodium* ^20^. STRING analysis demonstrated a confident clustering PPI among the candidate proteins, which supports the results obtained by comparative phosphoproteomic analysis (Fig. 1g). Importantly, PfCDPK1 was predicted to be involved intimately with several of its targets identified by phosphoproteomics such as GAP45 and IMC1g that were validated during the course of these studies.

### PfCDPK1 is involved in the phosphorylation of IMC proteins

Several IMC proteins, which are part of the actomyosin motor like GAP45 and Myosin A, or other IMC proteins such as IMC1c (ALV5), IMC1g (ALV4) associated with the IMC protein meshwork exhibited reduced phosphorylation upon PfCDPK1 knockdown (Fig. 1d). Previous studies have indicated that PfCDPK1 co-localizes with the Myosin Tail Interacting Protein, as well as Merozoite Surface Protein 1 (MSP1) in both schizonts and free merozoites^17^ suggesting that it is in close proximity to the IMC and plasma membrane. As mentioned above, PfGAP45-which was previously demonstrated to be an in vitro substrate of PfCDPK1^15, 21^-exhibited differential phosphorylation at S89 and S103 (Fig. 1d, Supplementary Data 2). Using an antibody which recognizes S103 phosphorylated PfGAP45^15^, we were able to demonstrate that PfGAP45 phosphorylation at this site is indeed reduced upon PfCDPK1 knockdown (Fig. 2a) and supported the phosphoproteomics data (Fig. 1d). In addition, two other IMC proteins PfIMC1g (PF3D7_1003600) (Fig. 2b) and PfIMC1c (PF3D7_0525800) were differentially phosphorylated (Fig. 1d). PfCDPK1 phosphorylated IMC1g in vitro (Fig. 2c). Importantly, metabolic labeling revealed that PfCDPK1 depletion resulted in reduced phosphorylation of PfIMC1g (Fig. 2d). These data strongly suggested that PfCDPK1 is a major kinase that phosphorylates IMC1g in the parasite. Moreover, PfCDPK1 exhibited localization in close proximity with IMC1g (Fig. 2e) in the parasite, which was reflected by overlapping fluorescence in IFA. In addition, the fact that IMC1g immunoprecipitated with PfCDPK1 suggested its interaction with the kinase in the parasite (Fig. 2f).

### Regulation of PfPKA by PfCDPK1

**Fig. 3:**
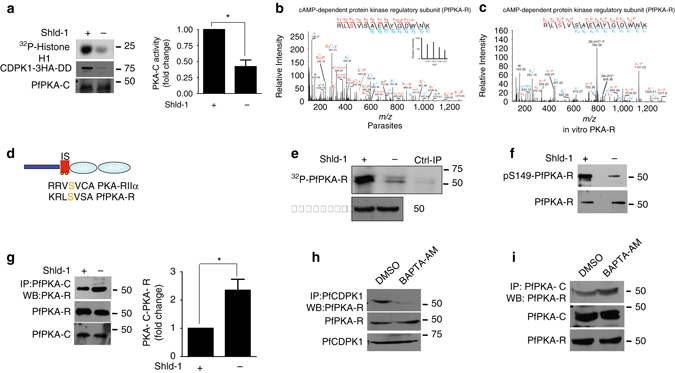
PfCDPK1 regulates PfPKA-C by targeting PfPKA-R. **a** PfPKA-C was immunoprecipitated after Shld-1 removal from PfCDPK1-3HA-DD parasites. PfPKA-C-IP was used to assay kinase activity using Histone H1 as substrate and its phosphorylation was detected by phosphorimaging and quantified by densitometry of the radiolabelled bands (Mean±s.e.m., * *n*=3, *p*<0.05, *t*-test). Western blotting was performed for PfPKA-R and CDPK1-HA on total parasite lysate. **b** and **c** The MS/MS spectra indicated that S149 exhibits reduced phosphorylation in the parasite in one of the replicates of phospshoproteomics experiments **b** PfCDPK1 kinase assays were performed using recombinant PfPKA-R in vitro and kinase assay mix was used for LC–MS/MS analysis, which revealed PfPKA-R to be phosphorylated at S149 in vitro **c** In addition, several other sites of PfPKA-R were found to be phosphorylated in vitro (Supplementary Fig. 3a). **d** PfPKA-R domain architecture indicating the differentially phosphorylated site (S149). The motif encompassing the phosphorylation site is indicated. The Inhibitory Segment (IS) sequence of PfPKA-R is compared with that of human PKA-RIIα. **e** PfCDPK1-3HA-DD parasites, at late trophozoite stage, were incubated with ^32^P-orthophosphate and harvested at late schizont/segmenter stage. PfPKA-R was immunoprecipitated and the IP was electrophoresed and radiolabeled proteins were detected by phosphorimaging. Western blotting with anti-PfPKA-R was performed on parasite lysate. A control IP was also performed using preimmune-sera on Shld-1 treated parasite lysate. **f** An antibody which specifically recognizes the S149-phosphorylated form of PfPKA-R was generated (Supplementary Fig. 3c). Western blotting was performed on lysates from PfCDPK1-3HA-DD parasites treated with Shld-1 or left untreated. An antibody, which recognizes both unphosphorylated and phosphorylated form of PKA-R, was used as a control for Western blotting (*bottom panel*). **g** Shld-1 was removed from PfCDPK1-3HA-DD parasite cultures as described above and schizont lysates were prepared after ~44 h. PfPKA-C was immunoprecipitated and PfPKA-C-IP was used for western blotting to detect PfPKA-R. Western blotting with indicated antibodies was also performed on total parasite lysates. *Right Panel*, Fold change in PfPKA-R associated with PfPKA-C was quantitated by densitometry after normalization with respect to PfPKA-R present in total parasite lysate (Mean±s.e.m., *n=4, *p*<0.05, *t*-test). **h** and **i** 3D7 schizonts were treated with BAPTA-AM to chelate intracellular calcium. Subsequently, PfCDPK1 (**h**) or PfPKA-C (**i**) was immunoprecipitated. PfCDPK1-IP and PfPKA-C-IP were immunoblotted with anti-PfPKA-R (**h** and **i**). Western blotting was also performed on parasite lysates using indicated antibodies

Recently published reports have hinted at a cross talk between cAMP and calcium signaling during invasion^22^. However, the underlying molecular mechanisms are poorly understood. cAMP mediates signaling via cAMP dependent protein kinase (PKA) in most organisms including the malaria parasite^23^. Therefore, we investigated the possibility of PfPKA regulation by PfCDPK1. To this end, PfPKA-C was immunoprecipitated and its activity was assayed upon PfCDPK1 knockdown. Interestingly, a significant decrease in its activity upon PfCDPK1 knockdown suggested that PfCDPK1 regulates PfPKA-C in the parasite (Fig. 3a).

As mentioned above, analyses of phosphoproteomic data revealed that the phosphorylation of PfPKA-R was down regulated at S149 upon PfCDPK1 knockdown in one of the replicates (Supplementary Data 1, Fig. 3b). It was worth noting that S149 belongs to K/RRxSxS motif (Fig. 3d), which is very closely related to one of the major PfCDPK1 target motifs indicated above (Fig. 1f) that were enriched among significantly altered phosphopeptides. In order to confirm if PfPKA-R is a substrate of PfCDPK1, in vitro kinase assays were performed using recombinant PfPKA-R. Indeed, PfCDPK1 phosphorylated PfPKA-R in vitro (Fig. 3c, Supplementary Fig. 3b). In addition to S149, in vitro phosphorylation of other sites of PfPKA-R was detected by LC–MS/MS studies (Fig. 3c, Supplementary Fig. 3a).

To confirm that PfPKA-R phosphorylation is compromised upon PfCDPK1 depletion, metabolic labeling of parasite proteins with ^32^P-labelled orthophosphate was followed by PfPKA-R-IP. These experiments revealed reduction in phosphate incorporation in PfPKA-R upon PfCDPK1 ablation, which indicated that PfCDPK1 is involved in PfPKA-R phosphorylation (Fig. 3e). An antibody against the S149-phosphorylated form of PfPKA-R, which mainly recognizes the S149 phosphorylated form of PfPKA-R was custom generated (Supplementary Fig. 3c). Using this antibody, a decrease in its phosphorylation at S149 was observed upon PfCDPK1 depletion (Fig. 3f). IFAs were performed to check if PfPKA-R and PfCDPK1 co-localized in late schizont parasites. PfCDPK1 exhibited co-localization with PfPKA-R (Supplementary Fig. 4c). In addition, co-immunoprecipitation of PfPKA-R with PfCDPK1 indicated their association in the parasite (Fig. 3h). PfPKA-R-PfCDPK1 interaction was also observed in parasites that overexpress PfPKA-R (Supplementary Fig. 4b).

S149, which is the target site of PfCDPK1 in the parasite, resides in the Inhibitory Segment (IS) of PfPKA-R (Fig. 3d). While cAMP binding to PKA-R is essential for dissociation of the R and the C subunits, subsequent phosphorylation of the IS contributes to this process and catalytic activation of the C subunit in the case of mammalian PKA^24^. The IS sequence is reasonably well conserved in mammalian and *Plasmodium* PKA-R and S149 aligns with corresponding site in PKA-R from other species^23^ (Fig. 3d). The impact of PfCDPK1 knockdown on PfPKA-R/PfPKA-C interaction was analyzed by performing co-immunoprecipitation experiments. PfPKA-R was pulled down with PKA-C in the presence of Shld-1 (Fig. 3g). Upon Shld-1 removal, there was a significant increase (Fig. 3g) in binding of these proteins, which suggested that PfCDPK1 may prevent interaction between the R and the C subunits of PfPKA. PfCDPK1 is activated by calcium, which induces conformational change that facilitates this process^7^. Co-IP experiments revealed that chelating intracellular calcium by BAPTA-AM resulted in a loss of PfPKA-R-PfCDPK1 interaction (Fig. 3h) suggesting that calcium-induced effects on PfCDPK1 are required for its binding to PfPKA-R. In contrast, BAPTA-AM treatment resulted in an increase in PfPKA R and C subunits (Fig. 3i) and a corresponding decrease in PfPKA activity (Supplementary Fig. 4a). Collectively, these data indicated that PfCDPK1 interacts and phosphorylates PfPKA-R in a calcium dependent manner, which may contribute to dissociation of PfPKA R and C subunits and promote the activation of PfPKA-C.

### A negative feedback loop between PfCDPK1 and PfPKA

**Fig. 4:**
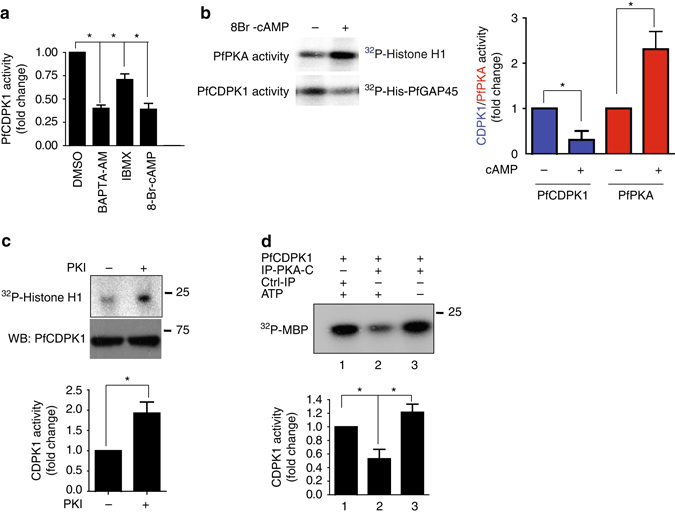
cAMP-dependent PfPKA-signaling may prevent PfCDPK1 activation via a negative feedback loop. **a** 3D7 schizonts were treated with DMSO, BAPTA-AM, IBMX or 8Br-cAMP for 3 h following which parasite lysates were prepared and PfCDPK1 was immunoprecipitated. PfCDPK1-IP was used to assay the kinase activity as described above using PfGAP45 as phosphoacceptor substrate. Fold change in activity was determined with respect to DMSO control (Mean±s.e.m., *n=3, *p*<0.05, *t*-test). **b** PfPKA-R-GFP parasites were left untreated (−) or treated with 8Br-cAMP (+) followed by immunoprecipitation with PfCDPK1 or PfPKA-C antisera and kinase assays were performed with the IP, as described above using recombinant PfGAP45 or Histone H1 as phosphoacceptor substrate and phosphorylation was detected by phosphorimaging and quantified by densitometry of the bands from the phosphorimage (*right panel*) (Mean±s.e.m., *n=3, *p*<0.05, *t*-test). **c**. 3D7 schizont-infected red blood cells were treated with 20 μM myristoylated PKI followed by immunoprecipitation of PfCDPK1 and kinase assay was performed as described above followed by quantitation of CDPK1 activity by densitometry of radiolabeled Histone H1 (Mean±s.e.m., *n=3, *p*<0.05, *t*-test). **d** To assess the effect of PfPKA on PfCDPK1 activity a two step assay was used. *Step 1:* PfPKA-C-HA was immunoprecipitated from PfPKA-C-HA parasites using anti-HA antibody. PfPKA-C-HA-IP (lane 2 and 3) or a control unrelated IP (lane 1) was incubated with recombinant PfCDPK1 in a kinase assay reaction in the presence (lane 2) or absence (lane 3) of unlabelled ATP. *Step 2:* Kinase assays were performed for PfCDPK1 obtained after Step 1 by using MBP as the phosphoacceptor substrate and radiolabelled ATP. The phosphorimage of the SDS-PAGE gel revealed that PfPKA-C-HA-IP inhibited PfCDPK1 activity when incubated with ATP (lane 2 vs. lane 3). *Lower Panel*, Densitometry was performed on radiolabeled bands to quantitate the phosphorylation and fold change in activity with respect to control (lane 1) is indicated. Data presented is Mean±s.e.m. of three independent experiments, *n=3, ANOVA, *p*<0.05)

In independent preliminary studies that were directed at understanding the effect of cAMP on calcium signaling, we noted that modulation of cAMP levels impacted PfCDPK1 activity. Strikingly, phosphodiesterase inhibitor IBMX, which sustains cAMP levels in the parasite^22^ and addition of 8Br-cAMP to parasites caused a significant decrease in PfCDPK1 activity (Fig. 4a). These data indicated that cAMP signaling may negatively regulate PfCDPK1 activity. The next logical step was to investigate the involvement of PfPKA in this process. We used parasites that overexpressed PfPKA-R as GFP fusion protein under AMA-1 promoter, which ensured its expression in schizont and segmenter stages. The overexpression of PfPKA-R sequesters PfPKA-C, which results in suppression of its activity and increasing cellular cAMP by addition of 8Br-cAMP to parasites resulted in elevation of the activity (Fig. 4b, *red bars*). In contrast, PfCDPK1 activity was significantly reduced (Fig. 4b, *blue bars*). A reverse experiment was also performed in which parasites were treated with a PKA inhibitor peptide PKI, which directly inhibits PfPKA-C^25, 26^. This inhibitor caused a significant increase in PfCDPK1 activity (Fig. 4c). Collectively, these data suggested that PfPKA-C may negatively regulate PfCDPK1 in the parasite.

Next, the direct effect of PfPKA-C on PfCDPK1 activity was assessed by performing in vitro kinase assays using recombinant PfCDPK1. Since recombinant PfPKA-C is catalytically inactive, a parasite line that over expresses PfPKA-C fused to 3xHA tag was used to immunoprecipitate PfPKA-C-HA and IP was used as a source of active PfPKA^27^. PfPKA-C-IP was incubated with PfCDPK1 in the absence or presence of unlabelled ATP, which was added to facilitate possible phosphorylation of PfCDPK1 by PfPKA-C. Subsequently, PfCDPK1 was separated from PfPKA-C-HA-IP. The activity of PfPKA-phosphorylated PfCDPK1 (+ATP, Fig. 4d, lane 2), or the unphosphorylated (-ATP, Fig. 4d, lane 3) was compared by performing assays using ^32^P-labelled ATP and MBP, which was used as a phosphoacceptor substrate for PfCDPK1. A significant decrease in PfCDPK1 activity was detected when PfPKA-C-HA-IP was added along with cold ATP (Fig. 4d, lane 2) in comparison to the reaction in which cold ATP was excluded from the first step of the assay (Fig. 4d, lane 3). In addition, a control IP of an unrelated protein failed to alter PfCDPK1 activity (lane 1). These results indicated that PfPKA-C may inhibit PfCDPK1 activity. The observed negative feedback loop may be mechanism via which PfPKA may down regulate or shut down PfCDPK1 in the parasite.

### PfCDPK1 may regulate RBC invasion by the parasite

**Fig. 5:**
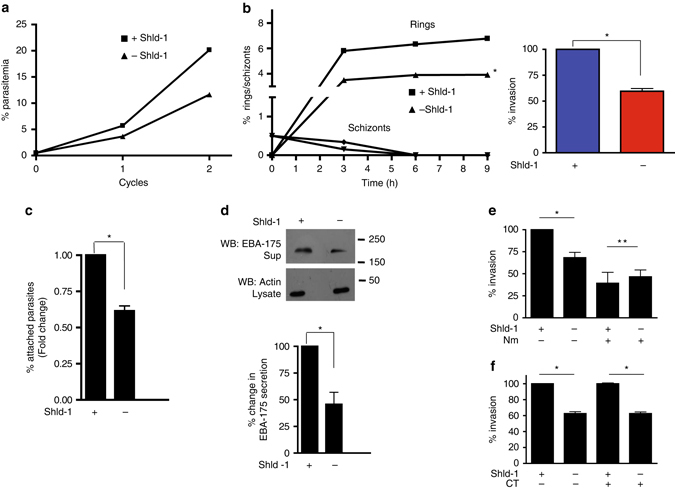
PfCDPK1 regulates invasion of host erythrocytes by *P. falciparum*. **a** Depletion of PfCDPK1 results in reduced growth of PfCDPK1-3HA-DD parasites. Synchronized PfCDPK1-3HA-DD parasites were cultured either in the presence or absence of Shld-1. % parasitemia was determined by counting at least 500 erythrocytes from Giemsa-stained thin blood smears which were prepared after every cycle, which indicated that parasites were mostly in ring forms. **b** PfCDPK1 may play a role in host cell invasion by *P. falciparum*. Schizonts±Shld-1 were incubated with fresh erythrocytes and after indicated time blood smears were prepared and the number of schizonts and rings were counted and represented as % infected erythrocytes. Shld-1 removal lead to a significant decrease in the number of rings, which were counted from thin blood smears (Mean ± s.e.m., *, *t*-test, *n*=4, *P*<0.05). *Right Panel*, % invasion was also determined by flow cytometry (Mean±s.e.m., *n*=4, *P<0.05, *t*-test). **c** PfCDPK1-3HA-DD parasites±Shld-1 were used for attachment assays performed using cytochalasin D (Cyt D). The number of attached merozoites were counted and data represents fold change in attachment with respect to Shld-1 treated parasites (100%) (Mean±s.e.m., *n*=3 *, *p*<0.05). **d** The release of EBA-175 by PfCDPK1-3HA-DD parasites±Shld-1 was determined by performing western blotting of culture supernatant. EBA-175 secretion was significantly reduced upon Shld-1 removal, which was quantitated by densitometry of the Western blot (*lower panel*, Mean±s.e.m., *n*=3 *, *t*-test, *p*<0.05). Actin blot was performed on the corresponding parasite lysate and used for normalization. **e** and **f** Shld-1 was withdrawn from parasites and invasion assays were performed using schizonts as described above except that in one case RBCs treated with either neuraminidase **e** or with chymotrypsin **f** were used for the assay. The number of rings formed, which reflected successful invasion, were determined. Data represents % invasion with respect to Shld-1 treated parasites (100%) (Mean±s.e.m., *n*=3 *, ANOVA, *p*<0.05, **p>0.05, NS)

PfCDPK1 seems to regulate a variety of parasite proteins, which possibly explains its importance for blood stage development^11, 12^. Efforts were made to understand the process it may regulate during blood stage development. To this end, the effect of PfCDPK1 knockdown on parasite growth was assessed by culturing parasites in the absence of Shld-1. A decrease in parasitemia was observed after each cycle, which was even more accentuated after the second cycle (~2 fold, Fig. 5a). Since decrease in parasitemia was observed mainly after each cycle and because PfCDPK1 is expressed mainly in schizonts^15^, a defect in egress or invasion was suspected. Next, we tested if the knockdown of PfCDPK1 had an impact on the invasion of host RBCs. To achieve this, Shld-1 was removed from ring stage parasites and schizonts were used for the invasion assays. Schizonts ruptured normally in the absence or presence of Shld-1 and were rarely observed after ~6 h of incubation with fresh erythrocytes suggesting that PfCDPK1 knockdown did not cause an apparent defect on egress. However, a significant decrease in the invasion, which was indicated by reduction in freshly formed rings, was observed in the case of Shld-1 deprived parasites (Fig. 5b). The invasion of RBCs by merozoites is a multi-step process initiated by the attachment to the RBC^28^. We tested if PfCDPK1 may regulate invasion by regulating the process of attachment. For this purpose, cytochalasin D (Cyt D) an inhibitor of actin polymerization, which allows attachment but prevents RBC entry^29, 30^ was used. Cyt D treated parasites from Shld-1+/− cultures were allowed to rupture and release merozoites that attached to erythrocytes. A significant decrease in parasites attached to erythrocytes was observed when PfCDPK1 was depleted (Fig. 5c) implicating PfCDPK1 in the host RBC attachment.

Invasion of host RBCs by *Plasmodium* is regulated by adhesins secreted by the parasite’s apical organelles called rhoptries and micronemes, which serve as ligands for receptors on host RBC surface^2, 31^. Therefore, the effect of PfCDPK1 knockdown on the secretion of major invasion ligands was tested. Western blotting of the culture supernatant containing secreted proteins revealed that the release of DBL domain protein EBA-175, which is a key invasion molecule^32^, was significantly reduced upon PfCDPK1 knockdown (Fig. 5d). However, another microneme protein AMA-1, which is involved in tight junction formation^33, 34^ did not exhibit a significant change. In addition, the exocytosis of rhoptry neck protein RH5^35^, which is involved in sialic acid-independent invasion, also remained largely unchanged in comparison to EBA-175 (Supplementary Fig. 5).

For the invasion of host erythrocytes, *P. falciparum* interacts with the sialic acid present on glycophorin A at the host surface via the EBL family of invasion ligands like EBA-175, which are released from the micronemes. In addition, it utilizes sialic acid independent pathways, which typically involve RH family members^31^. We probed if PfCDPK1 regulates both sialic acid-dependent and -independent pathways for invasion. The fact that PfCDPK1-3HA-DD parasites were generated in the D10 strain background allowed us to address this issue, as these parasites exhibit sensitivity towards neuraminidase^36^. When RBCs were treated with neuraminidase, it resulted in a significant decrease in invasion of control (Shld-1+) parasites (Fig. 5e, 1st bar vs. 3rd bar). While Shld-1 removal ablated invasion of untreated RBCs (1st bar vs. 2nd bar, Shld-1), the effect was less significant in the case of RBCs treated with Nm (3rd vs. 4th bar). These data suggested that PfCDPK1 may regulate invasion via a sialic acid-dependent receptor. While glycophorin A is resistant to chymotrypsin, there are several receptors for other parasite adhesins that are sensitive to chymotrypsin^31^. There was almost no difference in invasion efficiency of PfCDPK1-3HA-DD (Fig. 5f, 1st bar vs. 3rd bar) upon chymotrypsin treatment, which is consistent with parental D10 line being refractory to chymotrypsin treatment^36, 37^. PfCDPK1 depletion caused a similar defect in invasion of chymotrypsin-treated RBCs, as was the case with untreated RBCs (Fig. 5f, 1st bar vs. 2nd bar and 3rd bar vs. 4th bar). These data suggested that the observed invasion defects might be mainly due to the inability of the parasites to use the EBA-175/glycophorin A pathway.

### Regulation of invasion by PfCDPK1 and PfPKA

**Fig. 6:**
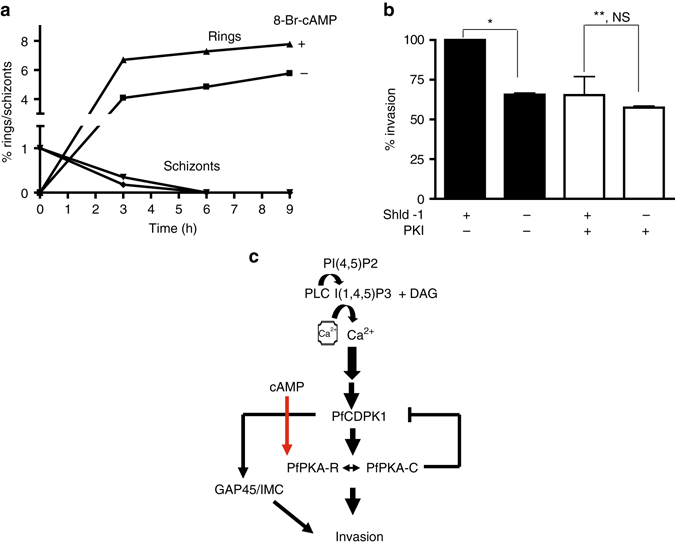
Regulation of invasion by PfCDPK1 and PfPKA. **a** The invasion of PKA-R-GFP schizonts in the absence (squares) or presence (triangles) of 8Br-cAMP was assessed by counting the number of rings and schizonts on Giemsa smears after indicated time as described above. A representative of three independent experiments is shown. **b** Shld-1 was withdrawn from the parasites and invasion assays were performed using schizonts as described above. In one set of experiments PKI was added for 3 h before addition of fresh RBCs for invasion. The number of rings formed were counted from Giemsa stained thin blood smears, which reflected successful invasion. Data represents % invasion with respect to Shld-1 treated parasites (100%) (Mean±s.e.m., *n*=2 *, ANOVA, *p*<0.05, **p>NS). **c** Model of PfCDPK1 mediated signaling in malaria parasite: PLC-mediated calcium release results in the activation of PfCDPK1 (Pushkar Sharma, unpublished results), which triggers phosphorylation of key substrates like IMC-glideosome proteins that include IMC1g, IMC1c, and PfGAP45. PfCDPK1 facilitates the activation of PfPKA-C by promoting its dissociation from PfPKA-R. PfCDPK1 may be turned off in the parasite via a negative feedback loop regulated by PfPKA. These events and the regulation of exocytosis of EBA-175 by PfCDPK1 signaling may contribute to the process of invasion

Previously, cAMP and PfPKA were implicated in microneme secretion^22^. Our studies suggested that PfPKA activity is regulated by PfCDPK1 (Fig. 3), therefore, attempts were made to see if this cross-talk may contribute to invasion. First, PfPKA-R-GFP parasites were used to confirm the role of PfPKA in invasion^22, 27^. An increase in the formation of rings was observed upon an increase in cAMP levels and schizont rupture remained almost unaltered (Fig. 6a), which was indicative of enhanced invasion and was consistent with a previous report^22^. The PKA-inhibitor PKI was also used to inhibit PfPKA in PfCDPK1-3HA-DD parasite-infected red blood cells. A marked reduction in invasion by PKI was observed (Fig. 6b, 1st bar vs. 3rd bar) in control Shld-1 treated parasites with normal PfCDPK1 expression implicating PfPKA in this process. While PfCDPK1 depletion (Shld-1) reduced invasion (Fig. 6b, 1st bar vs. 2nd bar), PKI was unable to cause an additional defect (4th bar vs. 2nd and 3rd bar). The significantly decreased sensitivity to invasion by the PKA-inhibitor when PfCDPK1 was depleted suggested that PfCDPK1-mediated regulation of PfPKA may contribute to the invasion process.

In summary, the present studies suggest PfCDPK1 may regulate invasion via phosphorylation and interaction with key proteins involved in IMC and glideosome function. Since PfCDPK1 and PfPKA regulate RBC invasion and PfCDPK1 is an upstream activator of PfPKA, it is reasonable to propose that their cross talk may contribute to the process of invasion (Fig. 6c).

## Discussion

While PbCDPK1 was considered to be non-essential for the growth of *P. berghei* ^10^, conditional knockdown of PbCDPK1 resulted in severe defects in ookinete differentiation and mosquito transmission^38^. Previous studies suggested that PfCDPK1 may be a critical enzyme, as its gene-disruption was not possible in *P. falciparum* ^11, 12^. Despite these studies, the function of PfCDPK1 in blood stage development of the human parasite had remained elusive. The present studies indicate that PfCDPK1 may regulate the invasion of host RBCs by *P. falciparum*, which corroborated well with its ability to regulate EBA-175 secretion. Interestingly, PfCDPK1 can complement TgCDPK3, which is its closest *Toxoplasma* homologue, and salvages the egress phenotype in *T. gondii* ^39^. The sialic acid -dependent invasion pathway, which involves interaction between DBL domain containing proteins like EBA-175 and the sialic acid moiety of glycophorins, was impaired upon PfCDPK1 depletion. However, invasion via chymotrypsin-resistant receptors like basigin^35, 40^ was not affected significantly. It will be surprising if PfCDPK1 specifically inhibits the sialic acid-dependent invasion events as it seems to target substrates, which may ubiquitously regulate the invasion process. It is possible that residual 30–40% expression of PfCDPK1 after Shld-1 removal may be sufficient for other pathways to operate. A recent study reported that knockdown of PfCDPK1 using the DD-FKBP domain did not alter parasite growth^13^. However, the knockdown obtained in these studies was nominal. GFP along with the DD domain was fused to PfCDPK1 instead of a HA-tag in these studies, which may have a bearing on the extent of PfCDPK1 degradation. In addition, the background parasites used were 3D7 in this case, which are largely insensitive to neuraminidase treatment and predominantly use the sialic acid-independent pathway^31^. The J-domain of PfCDPK1, which is inhibitory for kinase activity, caused parasite arrest in early schizogony and egress from parasites. It was rightly concluded from these studies that the J-Domain may also target proteins other than PfCDPK1^13^.

PfCDPK1 seems to regulate several proteins of the actomyosin motor and glideosome assembly, which connects the motor to the IMC. One of the features of network analysis was the connectivity exhibited by PfCDPK1 to proteins belonging to these complexes. Out of these, only PfGAP45^15, 18^ was previously implicated in PfCDPK1 signaling. Since almost all previous studies have been based on in vitro phosphorylation experiments, their association with and regulation by PfCDPK1 in the parasite remained to be established, which was possible by using PfCDPK1-3HA-DD parasites. PfGAP45 is phosphorylated at several sites other than S89 and S103. While mutation of some of these sites to A/D residues and overexpression of mutants did not alter glideosome function^15, 21^ it is likely that the cumulative effect of phosphorylation of these and other sites by kinases like PfPKG^41^ may regulate its function in *Plasmodium*. Other IMC proteins such as IMC1g/PfALV4 (PF3D7_0525800) and IMC1c/PfALV5 (PF3D7_10003600), which are alveolins^42^, were differentially phosphorylated upon PfCDPK1 knockdown. Both proteins are known to be heavily phosphorylated^41, 43^ and it will be interesting to dissect the role of their phosphorylation on IMC assembly and function. Interestingly, low molecular weight complex proteins RAMA-RAP1 were differentially phosphorylated upon PfCDPK1 depletion. Since PfCDPK1 does not seem to reside in rhoptries, it is possible that it may interact with these proteins in lipid rafts during trafficking, which was supported by a previous proteomics study that indicated^44^ the presence of these proteins in lipid rafts.

While a cross-talk between cAMP and calcium pathways has been reported in *Plasmodium* ^22, 25^, molecular interactions that guide it have largely remained unclear. We identified PfPKA-R as a substrate for PfCDPK1. The major phosphorylation site-S149, which is phosphorylated by PfCDPK1, resides in the inhibitory sequence, which lies upstream of the nucleotide binding domain (Fig. 3c). The crystal structure of the mammalian PKA complex indicates that IS docks into the active site of PKA-C rendering it to be inactive by engaging key residues^24^. Indeed, S149 phosphorylation of PfPKA-R was mediated by PfCDPK1 in the parasite, as suggested by use of a specific anti-pS149 antibody and co-immunoprecipitation of PfPKA-R and PfCDPK1 established their interaction in the parasite. PfCDPK1 knockdown results in enhanced interaction between R and C subunits of PfPKA-R, which corroborated well with the decrease in PfPKA-C catalytic activity. Therefore, it is reasonable to conclude that the phosphorylation and/or binding with PfCDPK1 promote the dissociation of PfPKA-R from PfPKA-C, which results in PKA-C activation. Given that K/RRxS motif that encompasses S149, may also be targeted by other kinases like AGC kinases and therefore, may be a key nodal site for cross talk of cAMP signaling with other signaling pathways as well.

A negative feedback loop via which PfCDPK1 activity can be turned off by PfPKA-C also emerged from present studies. Even though, we have not identified a PfPKA target site on PfCDPK1, it appears that PfPKA-C activity may contribute towards PfCDPK1 down regulation. A recent study suggested that PfPKG may phosphorylate PfCDPK1 at S64 and phosphorylation of this site was proposed to target PfCDPK1 to apical structures^41^. Our previous work demonstrated that S64 can be autophosphorylated by PfCDPK1 and mutation of this site does not cause a major change in its activity^7^. Therefore, the mechanism of PfCDPK1 regulation by PfPKA and PfPKG may be distinct, which will be interesting to elucidate. While this manuscript was under review, it was reported that the replacement of the gatekeeper residue in ATP-binding site attenuated PfCDPK1 activity and parasites expressing the gatekeeper mutant-PfCDPK1 were more sensitive to PfPKG inhibitor C2. Although direct demonstration was not provided, it was suggested that PfPKG or its downstream kinases might compensate for the loss of PfCDPK1 activity^45^, which will be an interesting proposition to test.

Previous reports have also suggested that PfPKA may regulate the secretion of microneme proteins like AMA-1 and EBA-175^22, 27^. We found mainly a defect in EBA-175 exocytosis and not in AMA-1, although, the possibility of residual ~30% PfCDPK1 being sufficient for AMA-1 release cannot be ruled out. Present studies highlight a novel hub involving PfCDPK1-PfPKA interaction for the cross-talk between calcium and cAMP pathways. Like PfCDPK1, PfPKA is also critical for blood stage development of *P. falciparum* ^12^. Inhibition with generic PKA inhibitors like H89 and PKI^25, 26^ implicated it in asexual development and invasion. Using PKI and transgenic PKA-R overexpressing parasites, we confirmed a role of PfPKA in RBC invasion. While PKI blocked invasion, its addition did not result in an additive effect upon PfCDPK1 knockdown, which hinted that PfCDPK1-mediated activation of PfPKA may be critical for invasion. While it is largely a correlation, it is indeed possible that PfCDPK1 may mediate EBA-175 secretion via PfPKA, as both these kinases regulate this process. Collectively, the present studies demonstrate the involvement of PfCDPK1 in regulating key modules like the IMC and cAMP signaling in the parasite, which might shed light on the mechanism via which it regulates host RBC invasion.

## Methods

### Reagents and antibodies

All the molecular biology reagents were purchased from Sigma-Aldrich. Oligonucleotides were procured form Sigma-Aldrich, restriction enzymes were obtained from New England Biolabs, USA. Commercially available antibodies were obtained from Santa Cruz Biotechnology (mouse anti- β-Actin (1:500, C4, sc-47778 HRP), rabbit anti-HA (1:500, Y-11, sc-805) and rabbit anti-GAPDH (1:500, FL 335, sc-25778)). Protein A/G plus agarose beads (sc-2003) were obtained from Santa Cruz Biotechnology. Anti-EBA-175 antibody was obtained from MR4 (1 : 1000, MRA-2). Other antibodies were kindly gifted by Dr. Chetan Chitnis, Institute Pasteur, Paris (1 : 1000, rabbit anti-AMA1), Prof. Alan Cowman, Walter & Eliza Hall Institute (1 : 2500, rabbit anti-RH5), Dr. Anthony A. Holder (1 : 2000 rabbit anti-GAP45). Shld-1 was purchased from Cheminpharma, LLC, USA. PKA Inhibitor PKI was obtained from Enzo life sciences (PKI_14-22_ amide, myristoylated), 8Br-cAMP, BAPTA-AM, Neuraminidase, trypsin, chymotrypsin were obtained from Sigma-Aldrich.

### Generation of transgenic parasites

For C-terminal tagging of PfCDPK1 locus with 3HA-DD, ~1.1 kb of the 3′end of the PfCDPK1 ORF (PlasmoDB ID PF3D7_0217500) without the stop codon was PCR amplified from *P. falciparum* genomic DNA (strain D10) with primers oJDD400 (5′-TAGGCGGCCGCTTAGTAACCGAATTTTATGAAGGTGGGG) and oJDD62 (5′- TAGCTCGAGTGAAGATTTATTATCACAAATTTTGTGCATCATG). The amplicon was ligated into 3HA-DD plasmid^46^ between the restriction sites *NotI* and *XhoI*, upstream and in-frame with the coding sequence of the 3HA and DD-tags^8, 37^. The PfCDPK1-3HA-DD plasmid, which has the human DHFR selection cassette, was prepared by using Qiagen Maxi prep kit. Approximately 100 µg plasmid DNA was transfected into D10 strain (WEHI) and selected using 5 nM WR99210 (gifted by Jacobus Pharmaceuticals). Integration of the plasmid was promoted by several cycles of drug treatment (~1 week) followed by culturing parasites without drug (~3 weeks). When parasites became resistant to WR99210 after a prolonged period off-drug, parasites were cloned by limiting dilution in a 96 well plate (nominal density, 3 parasites per 10 wells). The integration of 3HA-DD at the PfCDPK1 locus was confirmed by genotyping PCR, for which PCR primers were designed to amplify the endogenous locus, the 3′integration and the episome (see Supplementary Fig. 1). Furthermore, the 3′-integration amplicon was cloned and sequenced to confirm the integration at the desired locus.

The PfPKA-R overexpressing cell line was designed using the pARL-GFP vector^47^. The full length gene (PF3D7_1223100) was PCR amplified using Phusion DNA polymerase and PKA-RGFP_F and PKA-RGFP_R, KpnI/ AvrII digested and cloned into the digested pARL-GFP plasmid. The chimeric gene containing the open reading frame of PfPKA-R and GFP is driven by the late stage specific AMA1 promoter mimicking the expression profile of the endogenous *PfPKA-R* gene. The construct was sequenced and analyzed for accuracy.

### Parasite culture and transfections

*P. falciparum* asexual blood stages were cultured in O^+^ erythrocytes according to standard procedures^48^. In addition, 3D7 (MR4) were transfected with 100 µg of purified plasmid DNA, as described previously^49^. Positive selection of transfected parasites was achieved by addition of 10 nM WR99210, an antifolate that selects for the presence of the human *DHFR* gene. *P. falciparum* lines were cultured in complete RPMI 1640 medium with 0.5% albumax and gassed with 7% CO_2_, 1% O_2_ and 88% N_2_ at 37 °C. Synchronization of parasites was achieved by treatment with 5% sorbitol^50^. *P. falciparum* CDPK1-3HA-DD parasites were cultured in the presence of 2.5 nM WR99210 and 0.25 μM Shld-1. PfPKA-R-GFP and PfPKA-C-HA^27^ overexpressing parasite cultures were supplemented with 10 nM WR99210. PfCDPK1-3HA-DD parasites were generated in D10 background by transfecting plasmids for single cross-over homologous recombination. PfPKA-R-GFP overexpressing parasites were generated by transfection of 3D7 strain.

### Treatment of parasites with pharmacological reagents

Typically, schizont-infected RBCs were treated with IBMX (250 μM), BAPTA-AM (40 μM), 8Br-cAMP (100 μM), PKI (20 μM) unless indicated otherwise.

### Growth rate and invasion assays

For comparison of growth rate of the PfCDPK1-3HA-DD parasites in the presence or absence of Shld-1, parasites were synchronized using 5% sorbitol. The effect of Shld-1 removal on PfCDPK1-3HA-DD parasites was studied by splitting synchronized parasite cultures into two halves at ring stage. Shld-1 was withdrawn from one half and plating was done at equal parasitemia at the schizont stage. Parasite growth was quantified for 2–3 life cycles.

The parasite invasion assays were performed, as described earlier^4, 30^ with minor modifications. Invasion assays were performed by using schizonts from which Shld-1 was withdrawn for one cycle, as described above. Schizonts (~38–40 h.p.i) at 0.5–1% parasitemia were incubated with fresh RBCs and formation of new rings was monitored by examining Giemsa stained smears after 3–9 h and/or by performing FACS of SYBR Green stained parasites, which were fixed 30 h post invasion. FACS assay was performed using a slightly modified previous published protocol^51^. Briefly, ~300 µl parasite infected RBCs were washed thrice with PBS and were fixed with 4% paraformaldehyde for 30 min. After fixation, parasites were washed twice with PBS and stained with SYBR Green (1 : 5000 in PBS) for 1 h in dark. Cells were washed with PBS twice and resuspended in 800 µl of PBS and used for FACS using BD Accuri C6 Plus machine. Data were analyzed using FloJo software after excluding the autofluorescence from unstained cells by gating. To study the effect of Neuraminidase (Nm), ring infected RBCs were treated with 66 mU ml^−1^ Neuraminidase and 1 mg ml^−1^ Trypsin to prevent reinvasion, as described previously^32^ for 37 °C for 1 h. To study the effect of Chymotrypsin (CT), RBCs were treated with 1 mg ml^−1^ CT for 1 h at 37 °C, as described previously^36^. For performing invasion assays with PfPKA-R-GFP parasites, parasites were treated with 100 µM 8Br-cAMP 3 h prior to performing invasion assays.

### Parasite attachment assay

The attachment assays were performed as described earlier^30^ with slight modifications. Briefly, CDPK1-3HA-DD parasites were tightly synchronized, split into 2 × 15 ml (5% parasitemia, 5 % hematocrit) and from one half Shld-1 was withdrawn. The parasites were allowed to mature to schizonts (~ 40 h) and maintained in 50 U ml^–1^ of heparin for additional ~ 6 h. Subsequently, parasite-infected RBCs were plated in 6-well culture plates after washing with complete RPMI1640 along with fresh RBCs and treated with 1 μM cytochalasin D or DMSO (control). The plates were incubated in Ziploc bags, equilibrated with appropriate gas mixture and kept on a shaker at 80 rpm at 37 °C^30^. Thin blood smears were prepared after 8–12 h, which were used for staining parasite nuclei with DAPI. The numbers of attached parasites were counted by microscopically analyzing the DAPI stained parasites.

### Protein digestion and iTRAQ labeling

The work flow followed for these studies is described in Fig. 1c. The analysis was carried out on four independent biological replicates and one of the replicate was further processed and analyzed as technical duplicates. Therefore, the phosphorylation levels of proteins upon CDPK1 knockdown was studied in five independent analyses.

PfCDPK1-3HA-DD parasites were cultured in the presence or absence of Shld-1 and harvested at the late schizont stage, pellets were pooled and suspended in lysis buffer (8 M Urea, 1 mM Sodium fluoride, 1 mM sodium orthovanadate, 2.5 mM sodium pyrophosphate and 1 mM ß-glycerophosphate). Cells were lysed after multiple cycles of freeze thaw followed by sonication and centrifugation at 18,407 × g for 30 min. The supernatant was collected and protein was estimated using Bicinchoninic acid assay (Pierce, Waltham, MA, USA). An equal amount of protein (1.5 mg) from each case was reduced using DTT at a final concentration of 5 mM at 60 °C for 30 min and alkylated using 10 mM iodoacetamide for 10 min at room temperature kept in dark. Trypsin was added in a ratio of 1 unit of trypsin per 20 unit of sample and kept for overnight digestion at 37 °C. Buffer exchange was done using Sep-Pak C18 columns (Waters Corporation, Milford, MA, USA) and peptides were lyophilized. iTRAQ labeling of the peptides was carried out as per manufacturer’s instructions. Peptides from wild type parasites were labeled in technical replicates with 114 and 115 iTRAQ reporter ions and peptides from CDPK1 knockdown parasites were labeled with 116 and 117 reporter ions. Labeling of the peptides was checked and the four samples were pooled in equal amount and dried. One more biological replicate was processed in the same way, which was again analyzed as two technical replicates.

### Basic RPLC and phosphopeptide enrichment

The lyophilized peptides were reconstituted in bRPLC solvent A (10 mM triethylammonium bicarbonate, pH 8.5) and fractionated on Xbridge C18 5 um 250 × 4.6 mm column (Waters Corporation, Milford, MA, USA) using Agilent 1100 binary pump (Agilent Technologies, Santa Clara, CA, USA). The sample was resolved through reverse-phase liquid chromatography method using a gradient of 5 to 60% solvent B (10 mM triethylammonium bicarbonate, pH 8.5 in 95% Acetonitrile) with 1 ml flow rate per minute for over 60 min. A total of 96 fractions were concatenated into 12 fractions and vacuum dried. One tenth of each fraction was taken out for total proteomic analysis and the rest was further processed for TiO_2_ based enrichment of phosphopeptides. Titansphere beads (GL Sciences, Japan) were mixed with peptides in 1:1 ratio and incubated on rotor for 1 h at room temperature. Peptides were then washed with 80% ACN in 3% TFA and eluted using 4% ammonia solution followed by neutralization with 3% TFA. Eluted peptides were then dried and desalted using C_18_ stage tips and then subjected to LC-MS/MS analysis.

### LC-MS/MS analysis

Each fraction was reconstituted in 0.1% formic acid and analyzed on Orbitrap Fusion Tribrid mass spectrometer interfaced with Proxeon Easy-nLC 1000 system (Thermo Scientific, Bremen, Germany). The sample was first loaded onto a 2 cm long pre-column packed in-house with magic C18 AQ (MichromBioresources, Auburn, CA, USA). Peptides were resolved on an analytical column (75 µ × 15 cm, 3 µ particle and 100 Å pore size) using a linear gradient of 7 to 30% of solvent B (0.1% formic acid in 95% Acetonitrile) over 120 min. Both MS and MS/MS was acquired using Orbitrap mass analyzer and full scans were acquired with scan range of 350–1800 m/z and at a resolution of 120,000 at 200 m/z. The data were acquired in top speed mode with 3 s cycles, which would allow the instrument to continuously perform MS^2^ scans until the list of precursors diminish to zero or 3 s. Most intense precursor ions were selected and fragmented using higher-energy collisional dissociation (HCD) with 35% normalized collision energy. Fragment ions were detected in Orbitrap with mass resolution of 30,000 and AGC target value was set to 50,000 with maximum ion injection time of 200 ms. Singly charged ions were rejected and dynamic exclusion was set to 30 s. Internal calibration was carried out using lock mass option (m/z 445.1200025) from ambient air.

### MS data analysis

The mass spectrometry derived data were searched using Mascot and Sequest search engines using Proteome Discoverer 1.4 (Thermo Fisher Scientific, Bremen, Germany). Phosphopeptides enriched fractions and total proteome data from each replicate were searched against a combined Human and *P. falciparum* NCBI RefSeq protein database (version 65) with carbamidomethylation of cysteine residues, peptide N-terminal and lysine side chain of iTRAQ as fixed modification. Oxidation of methionine and phosphorylation of serine, threonine and tyrosine was selected as dynamic modifications. Trypsin was set as the protease and maximum of one missed cleavage was allowed. Precursor mass tolerance was set to 20 ppm and fragment mass tolerance of 0.1 Da was allowed. All PSMs were identified at 1% FDR. Posterior error probability was calculated for individual PSM using percolator, providing statistical confidence for each spectral match. If a given spectrum was assigned to different peptides by Mascot and Sequest, they were discarded and not considered for further analysis. The probability of phosphorylation for each site was calculated by the phosphoRS node in Proteome Discoverer. Only the phosphopeptides with >75% site localization were considered for further analysis. Phosphorylation fold change for each peptide was normalized with the total proteome level change and a normalized phosphorylation change of >0.75 fold was considered as significantly regulated.

### Gene ontology analysis

The gene ontologies for *Plasmodium falciparum* proteins were downloaded from PlasmoDB database^52^. Hypophosphorylated proteins that lacked appropriate GO annotation were assigned with relevant GO terminologies based on manual curation of the available literature. We merged our manually curated information with the available ontologies and carried out GO enrichment analysis on the list of hypophosphorylated proteins using FunRich tool^53^. The list of proteins identified in our analysis was selected as background and the hypophosphorylated proteins were provided as input. The fold- enrichment and *P* values associated with biological processes, molecular functions and cellular localizations are provided in Supplementary Data 2.

### Motif analysis

The list of hypophosphorylated sites with a width of 15 amino acids was subjected to motif analysis using motif-X tool^54^. A background of *P. falciparum* protein database was used for the analysis and occurrences threshold was set to default i.e. 20. A *P* value threshold of ≤ 1e-6 was used to identify enriched motifs.

### Protein-protein interaction networks prediction

The list of differentially phosphorylated proteins upon CDPK1 knockdown was submitted to STRING database (version 10.0) for protein–protein interaction analysis. Interactions with high confidence and score of >0.7 were considered as significant and considered for the study.

### Expression of recombinant proteins

For expression of PfPKA-C (PlasmoDB ID PF3D7_0934800), PfPKA-R (PlasmoDB ID PF3D7_1223100) and their mutants as GST fusion proteins, their ORFs were cloned in pGEX4T1 vector (GE Healthcare) using PCR primers PfPKA-R_F and PfPKA-R_R (PfPKA-R) and PfPKA-C_F and PfPKA-C_R (PfPKA-C). Primer sequences are provided in Supplementary Table 1. PfCDPK1 (PlasmoDB ID PF3D7_0217500) expression has been described previously^7^. For expression of PfIMC1g (PlasmoDB ID PF3D7_0525800), its ORF was cloned in pET28a (Novagen) vector using PCR primers PfIMC1g_F and PfIMC1g_R respectively. These and other 6xHis-tagged or GST-fusion recombinant proteins described in the manuscript were expressed and purified by using standard procedures using Ni-NTA or GSH-sepharose affinity matrices, respectively. Expression and purification of 6xHis-PfGAP45 has been described previously^15^. Protein concentration was determined by densitometry analysis of coomassie stained gels using NIH Image J software.

### Generation of antisera

Antisera was raised against PfPKA-C and PfPKA-R using KLH-conjugated synthetic peptides designed from their C-terminus following standard procedures in rabbits (2–3 months old, New Zealand) and mice (Balb/c, 4–6 week old) (Supplementary Fig. 6). PfPKA-C: H-(KLH) cys-VPYKPKYKNIFDSSNFE-OH; PfPKA-R: H-(KLH) cys-CDILHRNVENYKKVLNE-OH. Antisera against PfIMC1g was raised in mice using full length 6xHis PfIMC1g recombinant protein, which was expressed using standard procedures.

Antibody against PfPKA-R phosphorylated at S149 was custom generated by Antagene. Inc. (USA). For this purpose, a synthetic peptide with sequence

Cys- HFIQKKRL(p)SVSAEAYGDWNK or a control without phosphoserine was used. Cysteine at the N-terminus was added for KLH-conjugation. The phosphorylated and non-phosphorylated peptides were used to immunize rabbits over a 12-week schedule. Antisera were collected after 10 days of fourth immunization and antibody titers were determined by performing ELISA against the phosphorylated and the non-phosphorylated peptides. The antisera were enriched following a two-step phospho-peptide antibody purification method.

### Kinase assays

Protein kinase assays were performed using recombinant 6xHis-PfCDPK1 in a reaction buffer containing 50 mM Tris pH 7.4, 10 mM Magnesium Chloride, 1 mM Dithiothreitol, 100 µM CaCl_2_ and 100 μM γ^32-^P[ATP] 15μCi/reaction using recombinant substrates like PfGAP45, PfPKA-R, IMC1g. Reactions were carried out at 30 °C for 45 min and stopped by boiling them in SDS-PAGE loading buffer. The reaction mixture was electrophoresed on SDS-PAGE and phosphorylation of proteins was detected by phosphorimaging using a Fujifilm FLA-5000 scanner. For assaying activity of PfCDPK1 and PfPKA in the parasite, anti-PfCDPK1 or PfPKA-C antisera was used for immunoprecipitation and PfCDPK1-IP or PfPKA-IP was used for kinase assays as described above. For assaying PfPKA-C activity, a parasite line overexpresssing PfPKA-C-HA was used by immunoprecipitating using anti-HA antibody. The IP was subsequently used for kinase assays.

### Metabolic labeling of parasites

PfCDPK1-3HA-DD parasites were synchronized twice at the ring stage. Parasites were split in two halves after washing with complete RPMI 1640 media and Shld-1 was withdrawn from one of them. ~40 h post-invasion, parasites were washed twice with phosphate deficient RPMI 1640 medium (Hyclone) supplemented with 0.5% albumax (Gibco, Life Tech). These parasites were incubated with ^32^P orthophosphoric acid (1–2 mCi ml^–1^) till segmenters were formed. Parasite lysate was prepared in complete lysis buffer described below and immunoprecipitation was done using specific antibodies. The immunoprecipitate was electrophoresed on SDS-PAGE and labeled proteins were detected by phosphorimaging using a Fujifilm FLA-5000 scanner.

### Immunoblotting and immunoprecipitation

After various treatments with various pharmacological inhibitors, parasites were harvested by 0.05% saponin lysis at 4 °C for 30 min and were washed thrice with ice cold PBS and resuspended in complete lysis buffer containing 10 mM Tris pH 7.4, 100 mM sodium chloride, 5 mM ethylenediaminetetraacetic acid, 1% Triton X-100, 100 μM sodium orthovanadate, 20 μM β glycerophosphate, 1× protease inhibitor cocktail and 10% glycerol. Cell-free protein extracts were prepared from these parasites by syringe lysis and clearing by centrifugation at 15,871 × g at 4 °C for 30 min. After separation on 10% SDS-PAGE, lysate proteins were transferred to a nitrocellulose membrane. Immunoblotting was performed using specific antisera and blots were developed using SuperSignal West Pico, or Dura Chemiluminescence Substrate (Thermo Scientific) following manufacturer’s instructions.

For immunoprecipitation, 60–100 µg of lysate protein was typically used and incubated with relevant antibodies at 4 °C for 12 h followed by incubation with protein A+G plus agarose beads for 5 h. The beads were washed with cold complete lysis buffer and resuspended in 50 µl 1× kinase assay buffer for kinase assay and in 50 µl 1× SDS-PAGE loading buffer for western blotting. Immunoblots were cropped for presentation in main figures and full-sized images of some of the blots are presented in Supplementary Fig. 7.

### Detection of secreted proteins

PfCDPK1-3HA-DD parasites were synchronized and Shld-1 was withdrawn at the ring stage for 1–2 cycles, as described above. Fresh media was added to schizont/segmenters, which were allowed to burst to release merozoites. Subsequently, the culture was centrifuged at 500×g for 5 min to separate the free merozoites from the infected and uninfected RBCs. The culture supernatant containing merozoites was further centrifuged at 3300×g for 5 min to separate merozoite and secreted proteins released in the supernatant.

### Ethics statement

The animal experiments, which involved antisera generation, were performed at National Institute of Immunology, New Delhi with the approval of the Institute Animal Ethics Committee (IAEC) and prescribed guidelines were followed.

### Densitometry and statistical analysis

Image J (NIH) software was used to perform densitometry of western blots. The band intensity of the loading control was used for normalization. Statistical analysis was performed using Prism (Graph Pad software Inc USA). Data are represented as mean±Standard Error of Mean (s.e.m.) from three independent experiments, unless indicated otherwise and *p*<0.05 was taken as statistically significant.

### Data availability

The mass spectrometry-based proteomics data have been deposited to the ProteomeXchange Consortium (http://proteomecentral.proteomexchange.org) and can be accessed via the PRIDE partner repository with the data set identifier PXD005207.

All other relevant data are available from the authors upon request.

## Electronic supplementary material

Supplementary Information

Supplementary Data 1

Supplementary Data 2

Supplementary Data 3
