# Supplementary material for: PfCDPK1 mediated signaling in erythrocytic stages of Plasmodium falciparum
Source: Nat Commun. 2017 Jul 5;8:63. doi: 10.1038/s41467-017-00053-1 (PMC5498596; doi:10.1038/s41467-017-00053-1)
Supplement: Supplementary file 1 — Supplementary Information [file 41467_2017_53_MOESM1_ESM.pdf]

File name: Supplementary Information
Description: Supplementary figures and supplementary table.

File name: Supplementary Data 1
Description: a) Summary of phosphoproteomic and proteomic analyses of all replicates. b) Summary of phosphoproteomic and proteomic analyses of replicate 1. c) Summary of phosphoproteomic and proteomic analyses of replicate 2. d) Summary of phosphoproteomic and proteomic analyses of replicate 3. e) Summary of phosphoproteomic and proteomic analyses of replicate 4. f) Summary of phosphoproteomic and proteomic analyses of replicate 5.

File name: Supplementary Data 2
Description: a) List of hypophosphorylated sites among five replicates. b) Number of hypophosphorylated proteins under various biological processes. c) Cellular localization of the differentially phosphorylated peptides. d) Molecular functions of the differentially phosphorylated peptides. e) Motif analysis of the hypophosphorylated peptides. f) Theoretical and observed (highlighted in colors) fragment ions for the peptides illustrated in the figures.

File name: Supplementary Data 3
Description: String analysis for protein-protein interaction

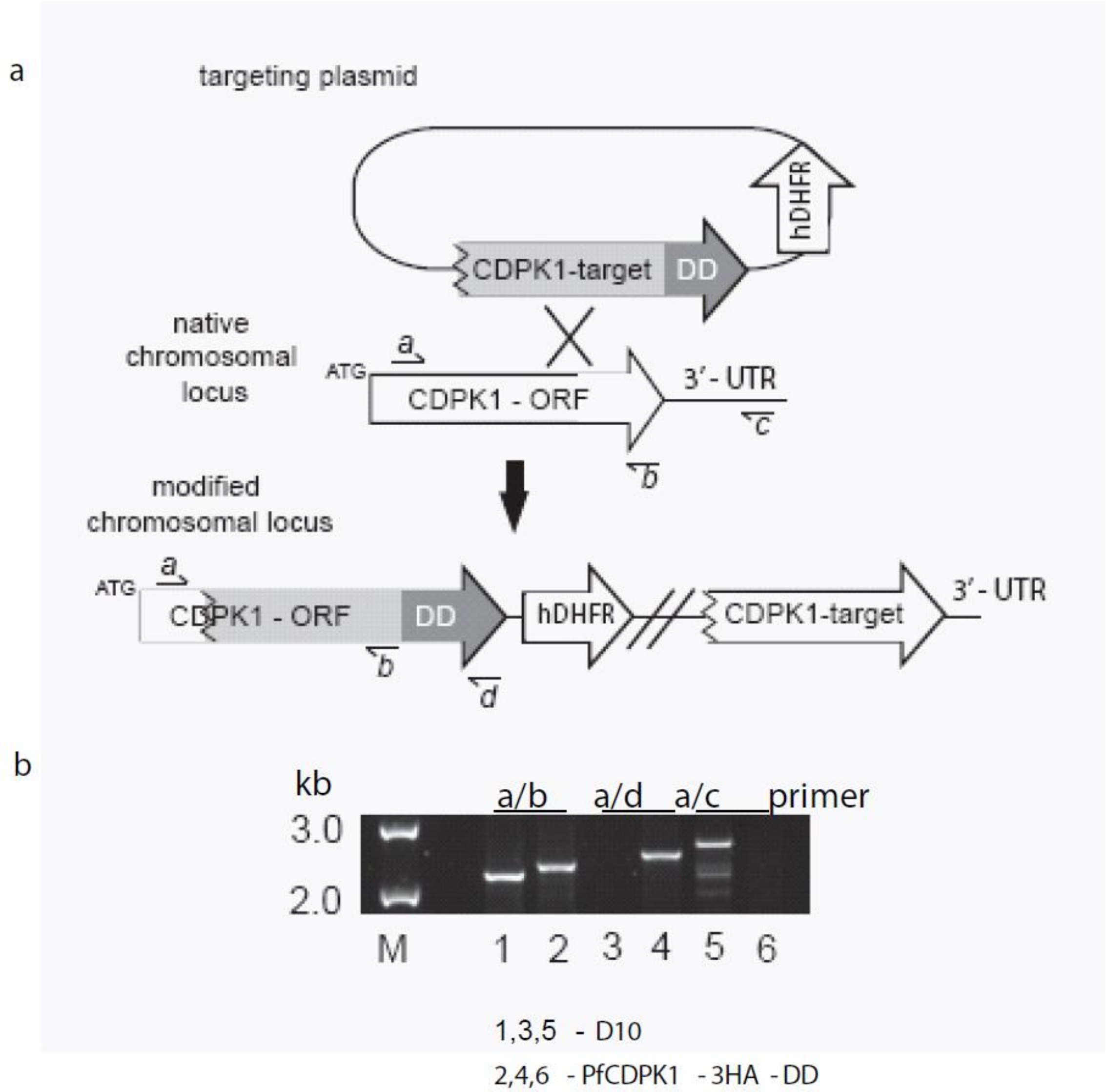

Supplementary Figure 1: (**a**). A schematic that illustrates the strategy for single crossover homologous recombination to generate PfCDPK1-3HA-DD at the chromosomal locus. Homology between the targeting plasmid and the 3'-end of endogenous PfCDPK1 mediates crossover. Sites for the ATG start codon in the PfCDPK1 ORF, the native PfCDPK1 3'-UTR, and human DHFR selectable marker are indicated. The binding sites for primers *a* (oAP417, 5'-GTTCACAAAGTTCAAACGTG), *b* (oJDD62 5'-

TAGCTCGAGTGAAGATTTATTATCACAAATTTTGTGCATCATG), *c* (oAP463, 5' - GACATGACCATATGTGTATTTC), *d* (oAP266, 5' – GGAAGGTGCGCCCGTC) to test integration of DD (see panel B) are also shown.

(**b**).The transgenic parasites were cloned by limited dilution and one of the clones obtained was analyzed by PCR for integration. PCR amplification from genomic DNA from the D10-parent (lanes 1, 3, and 5) or PfCDPK1-3HA-DD transgenic strain (lanes 2, 4, and 6), with primer pairs a/b (lanes 1 and 2, expected size 2215 bp), a/d (lanes 3 and 4, expected size 2357 bp), or a/c (lanes 5 and 6, expected size 2701 bp). The molecular weight marker (M) shows sizes of bands (kb). The amplification of a PCR product corresponding to the modified PfCDPK1-3HA-DD locus, which indicated successful 3'-integration was observed only in PfCDPK1-3HA-DD parasites (lane 4) but was absent in the parental D10 line (lane 3). The intact native PfCDPK1 was present in the D10 line (lane 5) but was absent from the PfCDPK1-3HA-DD parasites (lane 6). PCR products were also sequenced to confirm the integration at the expected locus.

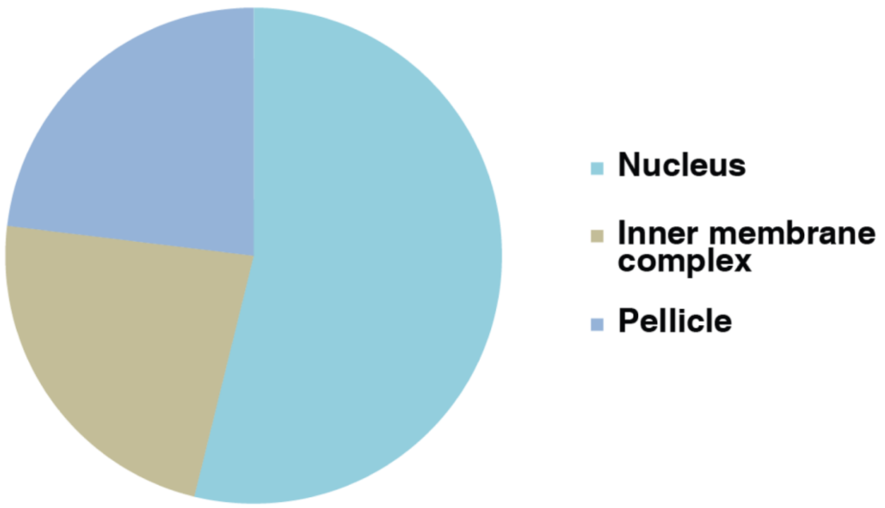

Supplementary Figure 2: Prediction of localization of PfCDPK1 target proteins.

Cellular localization information for the differentially phosphorylated proteins were obtained from Database for Annotation, Visualization and Integrated Discovery (DAVID) v6.8. The gene list was submitted to the tool followed by selection of gene functional classification.

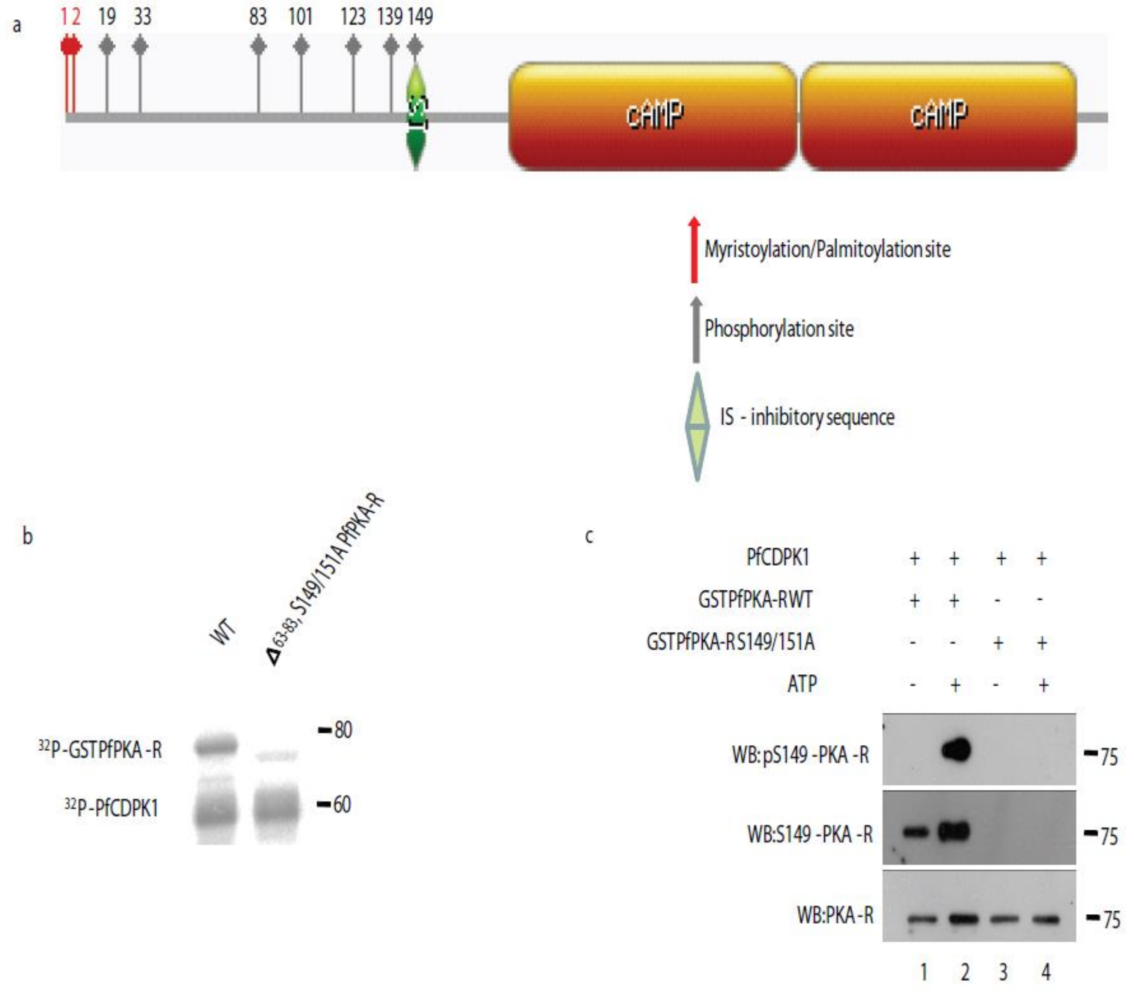

Supplementary Figure 3:

(**a**). Schematic for PfPKA-R indicating the location of various sites phosphorylated by PfCDPK1 in an *in vitro* kinase assay, which were identified by LC-MS/MS analysis. The lipid modification signal and the inhibitory sequence (IS) is also indicated.

(**b**). Recombinant PfPKA-R or a mutant ($\Delta_{63\text{-}83}$, S149A151A) in which residues 63-83 were deleted and S149/S151 was mutated to alanine was subjected to *in vitro* phosphorylation with PfCDPK1. The phosphorimaging of the SDS-PAGE gel revealed

that PfPKA-R is phosphorylated by PfCDPK1 and mutant exhibited only nominal phosphorylation.

(**c**). Custom antibodies were raised against phosphopeptide CHFIQKKRL-pS-VSAEAYGDWNK that spans the *in vivo* phosphorylated S149 or its unphosphorylated version CHFIQKKRLSVSAEAYGDWNK . To test these antibodies, recombinant PKA-R or its S149A mutant were phosphorylated with recombinant PfCDPK1 *in vitro* using ATP, which was excluded from the control samples (lane 1 and 3). The kinase assay mix was electrophoresed and immunoblotted with anti-pS149-PKA-R or anti-S149-PfPKA-R antisera. Anti-pS149 recognized only the phosphorylated PfPKA-R (lane 2) when it was phosphorylated with PfCDPK1 confirming the specificity of this antibody for S149 phosphorylated form of PfPKA-R.

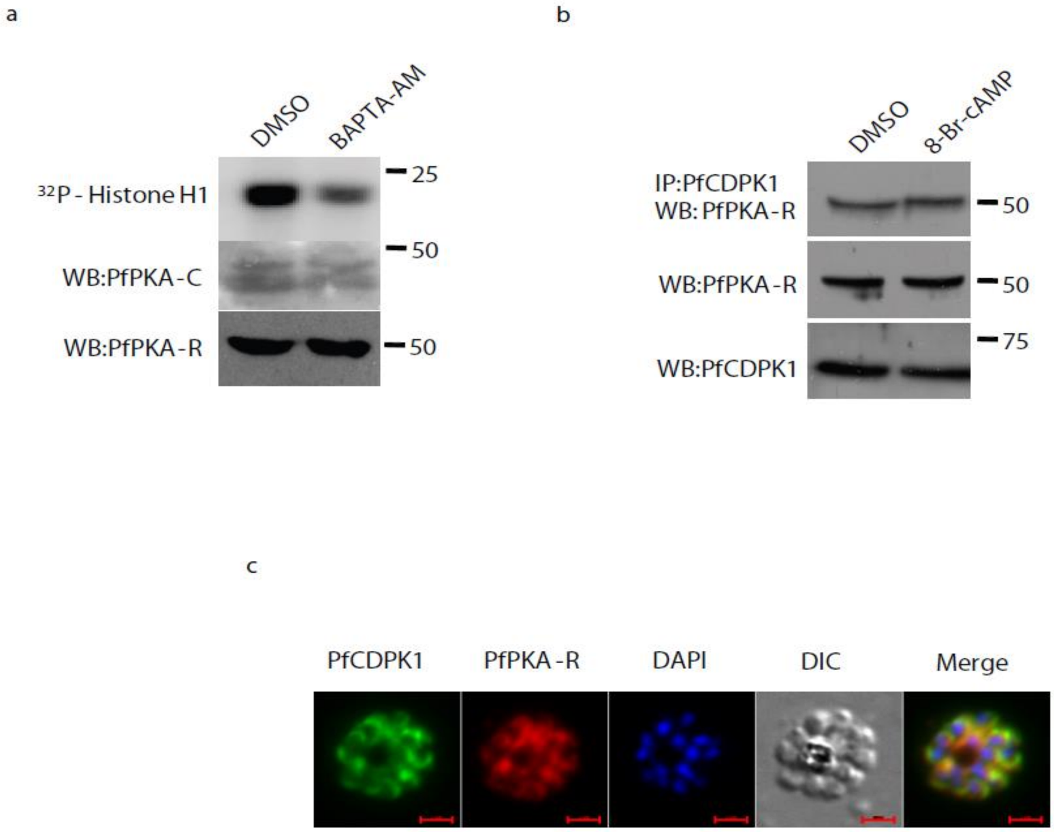

Supplementary Figure 4:

(**a**). 3D7 schizonts were treated with BAPTA-AM to chelate intracellular calcium. Subsequently, PfPKA-C was immunoprecipitated and PfPKA-C-IP was used to assay associated kinase activity using Histone H1 as phosphoacceptor substrate as described

above for figures. Western blotting was performed on whole cell lysates using indicated antibodies. Scale bar is 2 µm.

(**b**). PfPKA-R-GFP overexpressing schizonts were treated with 8-Br-cAMP. Subsequently, PfCDPK1 was immunoprecipitated and PfPKA-R associated with PfCDPK1 was detected by Western blotting. Western blotting was performed on whole cell lysates using indicated antibodies.

(**c**). IFA was performed on 3D7 parasites using anti-PfCDPK1 and anti-PfPKA-R antisera. Significant co-localization was observed between the two proteins.

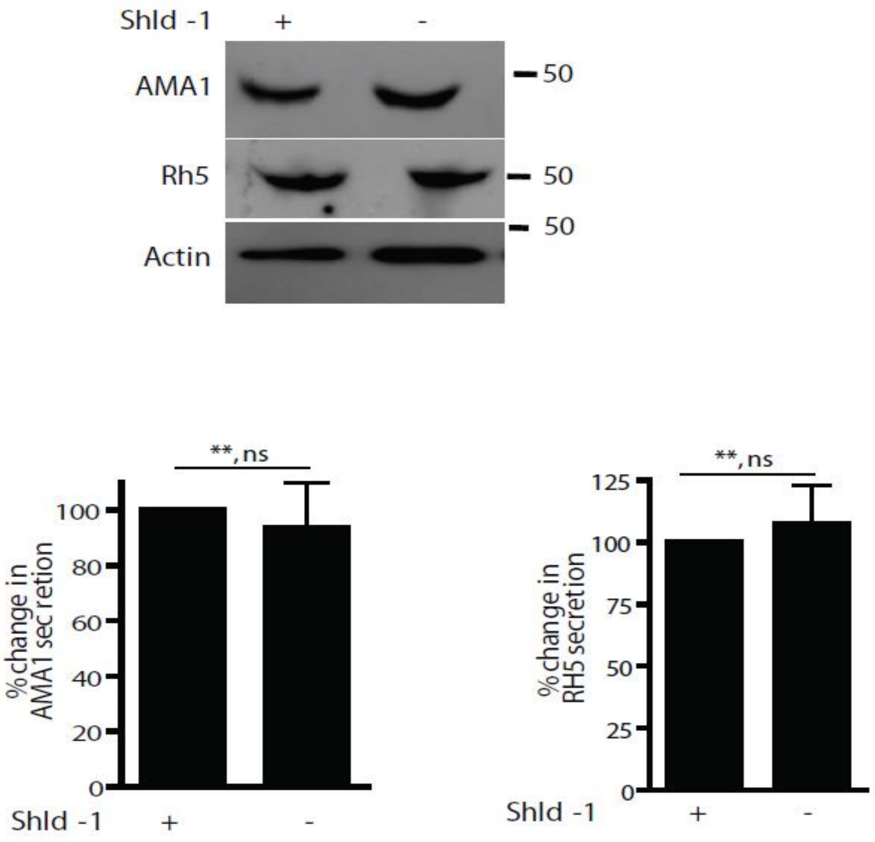

Supplementary Fig. 5: The release of RH5 and AMA-1 by PfCDPK1-3HA-DD parasites ± Shld-1 was determined by performing Western blotting of culture supernatant. The secretion of these proteins was quantitated by densitometry of the Western blot (lower panel, SEM +/-SE, n=3 **, t-test, P>0.05). Actin blot was performed on the corresponding parasite lysate and used for normalization.

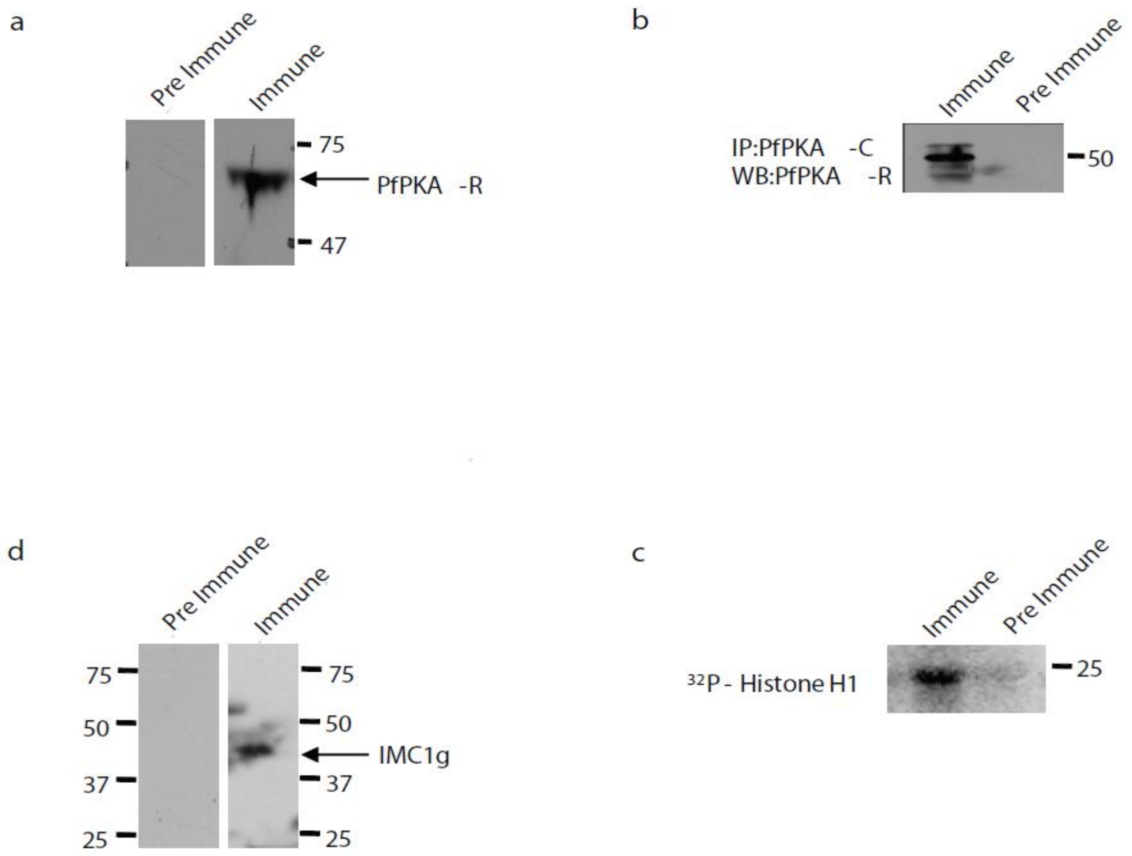

Supplementary Figure 6:

Antisera was raised against either synthetic peptides against PfPKA-R and PfPKA-C (**a** and **b**) or recombinant IMC1g (**d**) by immunizing mice (**a** and **d**) or rabbits (**b**).

b and **c**. Immunoprecipitation was performed using anti-PfPKA-C antisera or control pre-immune antisera. IP was used for Western blotting with anti-PfPKA-R antibody (**b**) or *in vitro* kinase assays (**c**) using Histone H1 as phosphoacceptor substrate as described earlier.

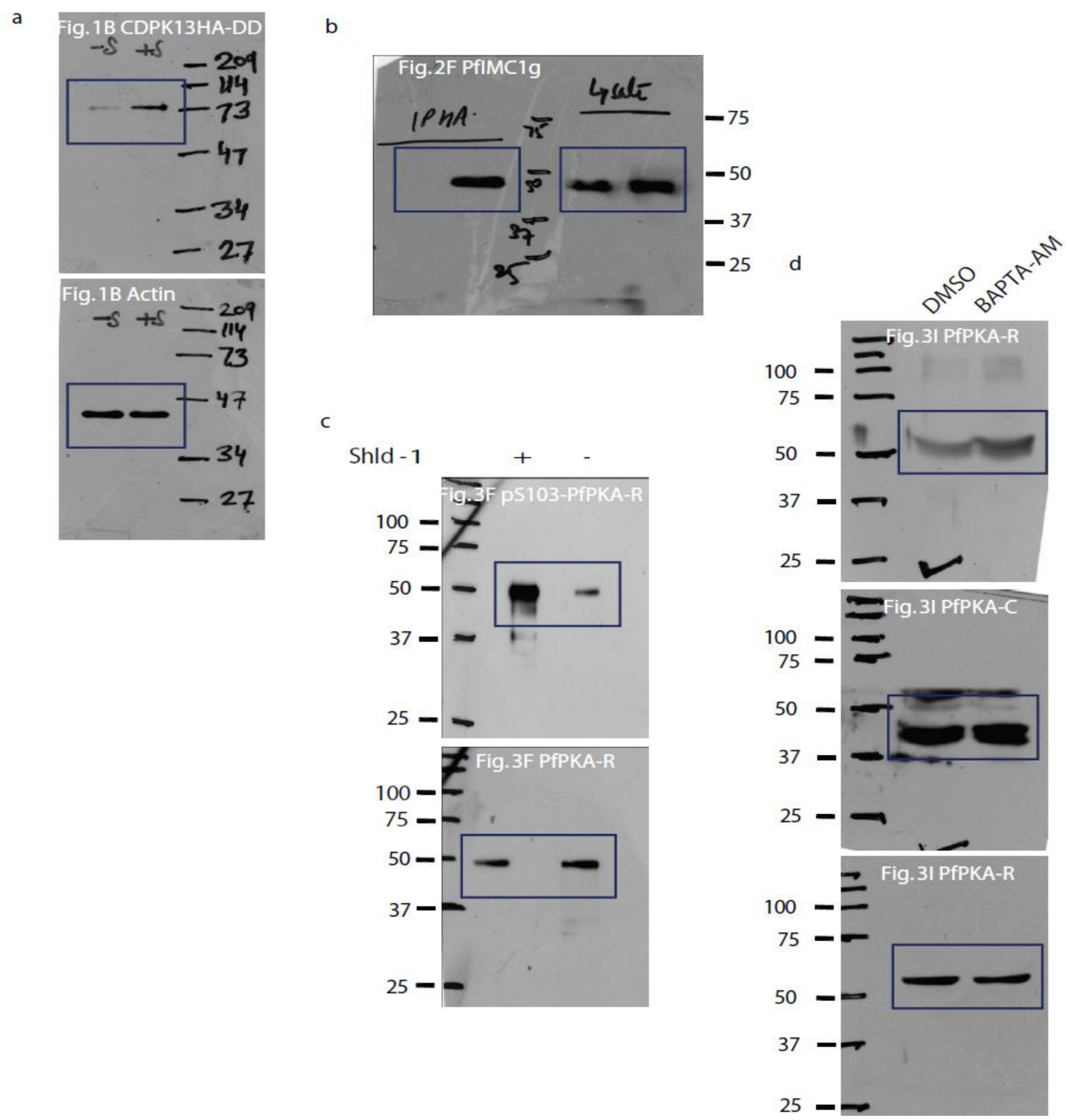

Supplementary Figure 7:

Full–sized immunoblots for Western blots provided in Figure 1b (**a**), Figure 2f (**b**), Figure 3f (**c**) and Figure 3i (**d**) are shown. The blue box on each immunoblot shows the cropped area displayed in the other figures.

Supplementary Table 1: PCR Primers used in the studies*:

Oligo	Forward (5'-3')	Reverse (5'-3')
PfPKA-R-pGEX	**PfPKA-R_F**<br>CGCGGATCCATGGGCAATG TGTGCACATGGAGACAAG GG AAAG	**PfPKA-R_F**;<br>CCGCTCGAGATTTTCATCAA TACAAGTTGTATCCAAACCT AA TTCG
PfPKA-C-pGEX	**PfPKA-C_F;**<br>CGCGGATCCCAGTTTATTA AAAATTTGCAG	**PfPKA-C_F;**<br>CGAGCGGCCGCACTACCAAT CATAAAATGG
PfPKA-R, Δ63-83 S149/S151AST	**Δ63-83, S149A151A_F;**<br>AGATGATAGATACAAATTT TCCGAGATATTAGATGGAT TGGATTA	**Δ63-83, S149A151A_R;**<br>TAATCCAATCCATCTAATAT CTCGGAAAATTTGTATCTAT CATCT
PfIMC1g	**PfIMC1g_F;**<br>CGCGGATCCATG TGTTCT ACAAATAAGAAT	**PfIMC1g_R;**<br>CGA GCGGCCGC TTAAGC ATATGAACAGTAAAG
PfPKA-R-GFP-pARL	**PKA-RGFP_F**;<br>CCGGGGTACCATGGGCAAT GTGTGCACATGGAGACAA GGGAAAGAAAAAG	**PKA-RGFP_R**;<br>CCGGCCTAGGATTTTCATCA ATACAAGTTGTATCCGGCC
PfCDPK1-3HA-DD	**oJDD400**;<br>TAGGCGGCCGCTTAGTAAC CGAATTTTATGAAGGTGGG G	**oJDD62:**<br>TAGCTCGAGTGAAGATTTAT TATCACAAATTTTGTGCATC ATG

* Some of the primer sequences have been mentioned in text and figure legends.
